# Clinical features and outcomes of pregnant women with COVID-19: a systematic review and meta-analysis

**DOI:** 10.1186/s12879-020-05274-2

**Published:** 2020-08-03

**Authors:** Yi-jie Gao, Lei Ye, Jia-shuo Zhang, Yang-xue Yin, Min Liu, Hong-biao Yu, Rong Zhou

**Affiliations:** Department of Obstetrics and Gynecology, West China Second University Hospital, Sichuan University, Key Laboratory of Birth Defects and Related Diseases of Women and Children (Sichuan University) of Ministry of Education, Postal address: No. 20, Section 3, South Renmin Road, Cheng Du, Sichuan province 610041 P. R. China

**Keywords:** SARS-CoV-2, COVID-19, Clinical features, Pregnancy outcomes, Pregnant women, Meta-analysis

## Abstract

**Background:**

The recent COVID-19 outbreak in Wuhan, China, has quickly spread throughout the world. In this study, we systematically reviewed the clinical features and outcomes of pregnant women with COVID-19.

**Methods:**

PubMed, Web of Science, EMBASE and MEDLINE were searched from January 1, 2020, to April 16, 2020. Case reports and case series of pregnant women infected with SARS-CoV-2 were included. Two reviewers screened 366 studies and 14 studies were included. Four reviewers independently extracted the features from the studies. We used a random-effects model to analyse the incidence (P) and 95% confidence interval (95% CI). Heterogeneity was assessed using the I^2^ statistic.

**Results:**

The meta-analysis included 236 pregnant women with COVID-19. The results were as follows: positive CT findings (71%; 95% CI, 0.49–0.93), caesarean section (65%; 95% CI, 0.42–0.87), fever (51%; 95% CI, 0.35–0.67), lymphopenia (49%; 95% CI, 0.29–0.70), coexisting disorders (33%; 95% CI, 0.21–0.44), cough (31%; 95% CI, 0.23–0.39), fetal distress (29%; 95% CI, 0.08–0.49), preterm labor (23%; 95% CI, 0.14–0.32), and severe case or death (12%; 95% CI, 0.03–0.20). The subgroup analysis showed that compared with non-pregnant patients, pregnant women with COVID-19 had significantly lower incidences of fever (pregnant women, 51%; non-pregnant patients, 91%; *P <* 0.00001) and cough (pregnant women, 31%; non-pregnant patients, 67%; *P <* 0.0001).

**Conclusions:**

The incidences of fever, cough and positive CT findings in pregnant women with COVID-19 are less than those in the normal population with COVID-19, but the rate of preterm labor is higher among pregnant with COVID-19 than among normal pregnant women. There is currently no evidence that COVID-19 can spread through vertical transmission.

## Background

Unexplained clusters of pneumonia cases related to the south China seafood wholesale market were reported in Wuhan City, Hubei Province, China, in December 2019 [1]. Chinese scientists isolated the novel coronavirus from patients, sequenced the genome, and found that the genetic sequence of the virus was at least 70% similar to that of human severe acute respiratory syndrome coronavirus (SARS-CoV). The World Health Organization (WHO) named the novel coronavirus 2019-nCoV [2], also called severe acute respiratory syndrome coronavirus 2 (SARS-CoV-2), which causes coronavirus disease 2019 (COVID-19) [3]. As of June 6, 2020, more than 84,000 cases have been confirmed in China, and 6,728,000 cases have been confirmed worldwide [4].

Both SARS-CoV-2 and SARS-CoV are β-coronaviruses. The mortality rate of SARS-CoV infection is 10%, including a mortality rate of 25% for maternal infection [5]. The clinical outcomes of pregnant women are worse than those of non-pregnant women. To date, clinical data on pregnant women with SARS-CoV-2 are very limited. Therefore, we conducted this systematic review and meta-analysis to assess the clinical features and pregnancy outcomes of pregnant women with COVID-19 to help formulate clinical treatment strategies.

## Methods

### Search strategy

The protocol for the meta-analysis was based on the MOOSE (Meta-analysis Of Observational Studies in Epidemiology) checklist [6] and EQUATOR Reporting Guidelines (Preferred Reporting Items for Systematic Reviews and Meta-Analyses) [7]. We only conducted a literature review; thus, ethics approval was not required.

We systematically searched the literature in the PubMed, Web of Science, EMBASE, and MEDLINE databases. The retrieval period was from January 1, 2020, to April 16, 2020. The search keywords were as follows: ((COVID-19) OR (2019 novel coronavirus infection) OR (COVID19) OR (coronavirus disease 2019) OR (coronavirus disease-19) OR (2019-nCoV disease) OR (2019 novel coronavirus disease) OR (2019-nCoV infection) OR (SARS-CoV-2) OR (Wuhan coronavirus) OR (Wuhan seafood market pneumonia virus) OR (COVID19 virus) OR (COVID-19 virus) OR (coronavirus disease 2019 virus) OR (SARS-CoV-2) OR (SARS2) OR (2019-nCoV) OR (2019 novel coronavirus)) AND ((Pregnancy) OR (Pregnancies) OR (Gestation) OR (Pregnant Women) OR (Pregnant Woman) OR (Woman, Pregnant) OR (Women, Pregnant)). The literature search had no language restrictions. We used the Endnote X7 library (Clarivate Analytics, Philadelphia, PA, USA) to remove duplicate citations and manage the references. We hand-searched the bibliographies of the retrieved papers for additional references.

### Selection criteria

#### Inclusion criteria

1. Case reports, case series and observational studies of pregnant women with COVID-19.
2. Description of the clinical features and/or outcomes of the patient and the foetus/new-born.

#### Exclusion criteria

1. Literature that has been republished;
2. Article types including authors’ replies, editorials, guidelines;
3. Case reports, case series and observational studies that have a number of cases less than 5;
4. Literature with incomplete or missing data.

### Data extraction and analysis

#### Data extraction

The two reviewers (LY and JS.Z) independently screened the literature based on the search strategy, inclusion criteria and exclusion criteria and extracted relevant data. When the reviewers’ opinions were inconsistent, they sought the opinion of the third reviewer (YJ.G) or negotiated solutions.

#### Quality assessment

Four reviewers (YJ.G, YX.Y, ML, and HB. Y) independently extracted the following features of the literature, listed in the study characteristics section: first author, publication date, study date, the number and age of patients and the number of severe cases or deaths, fever, cough, lymphopenia, positive CT findings, coexisting disorders, preterm labor, caesarean section, fetal distress, neonatal asphyxia or neonatal death or stillbirth, neonatal infection, and virus in the breast milk. They also evaluated the quality of the literature using the Institute of Health Economics (IHE) case series methodological quality evaluation tool [8], which evaluated 8 areas of the literature: (1) Research purpose, (2) Research population, (3) Intervention and joint intervention, (4) Outcome measures, (5) Statistical analysis, (6) Results and conclusions, (7) Conflict of interest and funding sources, and (8) New entry. Of the aforementioned 20 items that were extracted and evaluated for each study, studies that provided information related to 14 (70%) or more of the items were considered to be of acceptable quality.

#### Statistical analysis

All calculations were performed with Review Manager software (version 5.3, Nordic Cochrane Centre) and were guided by the previous work, Implement Meta-Analysis with Non-Comparative Binary Data in RevMan Software [9]. The *I*^*2*^ statistic was used to assess heterogeneity among the studies. An *I*^*2*^of less than 25% indicated low heterogeneity, 25–50% indicated medium heterogeneity, and more than 50% indicated high heterogeneity. Because of the high heterogeneity of this study, we used a random effects model to pool the study-specific frequencies and 95% confidence intervals (95% CIs) of the clinical features or outcomes. A *P* < 0.05 using the Cochran’s Q test was considered statistically significant. Funnel plots were used to assess the publication bias. A subgroup analysis was used to assess the sensitivity.

## Results

### Study selection

**Fig. 1:**
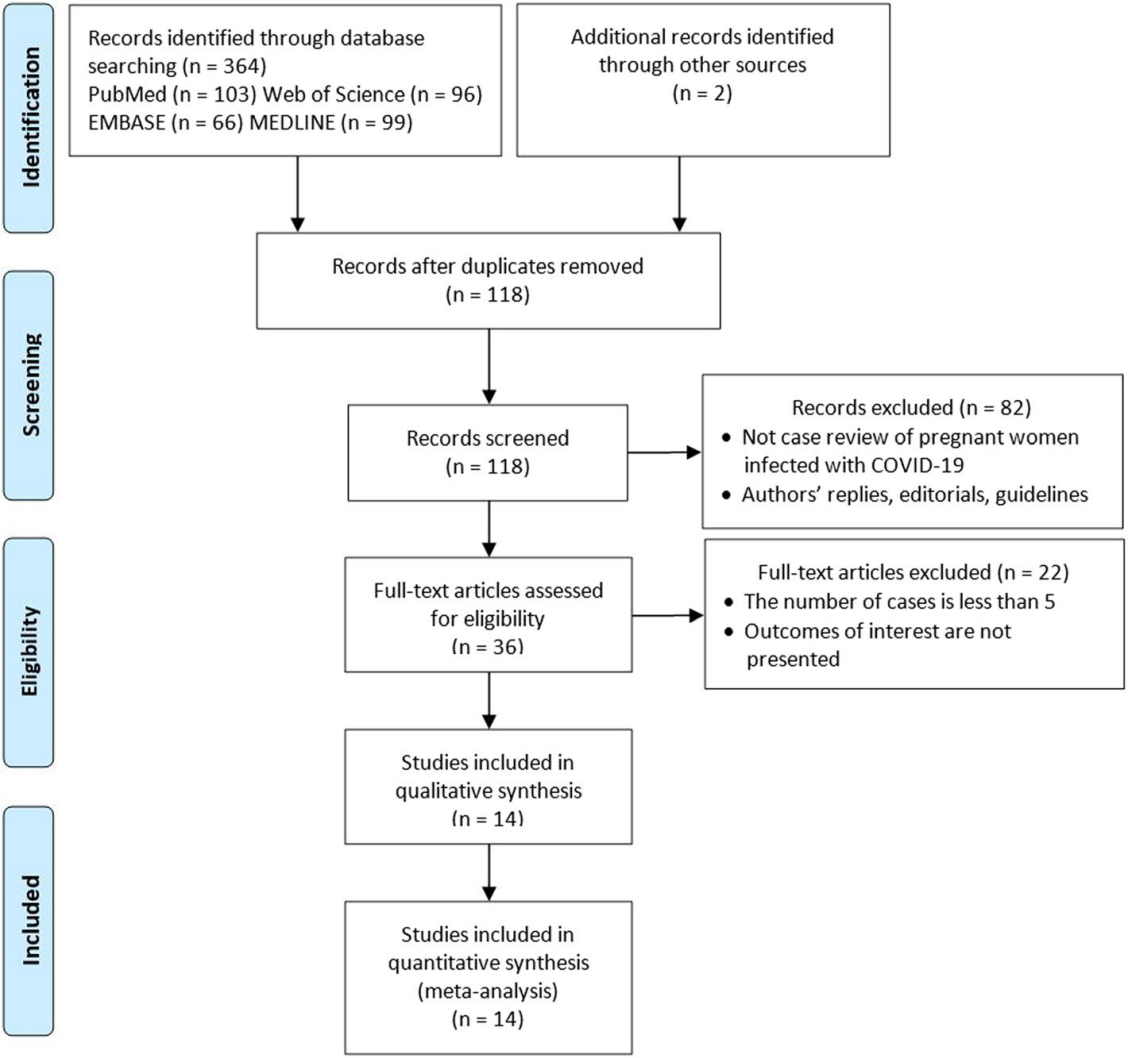
PRISMA flow diagram of study process

A total of 364 relevant documents were retrieved by the search methods above, including 103 articles from PubMed, 96 articles from Web of Science, 66 articles from EMBASE, and 99 articles from MEDLINE. We hand-searched the bibliographies of the retrieved papers, and 2 additional articles were included. After the removal of 248 duplicate documents, 82 papers were deemed ineligible after the title and abstract screening, and 22 papers were excluded after further screening through reading the full text. After the exclusion of all 352 unqualified studies, a total of 14 retrospective case analyses were included in this meta-analysis [10–23]. The process of the study selection is illustrated in Fig. 1.

### Study characteristics

We extracted the features of the literature above. The study included 236 pregnant women with laboratory-confirmed COVID-19 from December 8, 2019, to April 4, 2020, of whom 160 were in China and 76 were in America. The characteristics of the included literature are presented in Table 1.

**Table 1:** Characteristics of the studies included in the meta-analysis

Author and publication date	Study date (year, month, day)	Patients (No.)	Age (year)	Severe cases or deaths (%)	Fever (%)	Cough (%)	Lymphopenia (%)	Positive CT findings (%)	Coexisting disorders (%)	Preterm labor (%)	Caesarean section (%)	Fetal distress (%)	Neonatal asphyxia or neonatal death or stillbirth (%)	Neonatal infection (%)	Virus in breast milk (%)
Breslin N 2020.4.9 [10]	2020.3.13–2020.3.27	43^(a)^	26.9	14	33	44	–	9	42	–	19	–	–	0	–
Chen H 2020.2.12 [11]	2020.1.20–2020.1.31	9	29.9	0	78	44	56	89	33	44	100	22	0	0	0
Chen R 2020.3.16 [12]	2020.1.30–2020.2.23	17	29.4	0	24	24	29	100	47	18	100	0	0	0	–
Chen S 2020.3.28 [13]	2020.1.20–2020.2.10	5	28.8	0	100	20	80	100	60	0	40	0	0	–	–
Khan S 2020.4.8 [14]	2020.1.25–2020.2.15	17	29.3	–	18	35	24	29	29	18	100	0	0	12	–
Li N 2020.3.30 [15]	2020.1.24–2020.2.29	16	30.9	0	25	0	13	94	69	25	88	13	0	0	–
Liu D 2020.3.7 [16]	2020.1.20–2020.2.10	15	32	0	87	60	80	100	13	–	67	–	0	–	–
Liu H 2020.3.11 [17]	2020.1.27–2020.2.14	16	30	0	44	38	56	81	20	–	–	0	0	–	–
Liu Y 2020.2.27 [18]	2019.12.8–2020.2.25	13	29.7	8	77	15	–	–	8	46	77	23	8^(b)^	0	–
Sutton D 2020.4.13 [19]	2020.3.22–2020.4.4	33^(a)^	–	–	21	–	–	–	–	–	–	–	–	–	–
Wu X 2020.4.8 [20]	2019.12.31–2020.3.7	23	29	0	17	26	–	35	35	13	78	–	0	0	–
Yang H 2020.4.12 [21]	2020.1.20–2020.3.5	13	30.2	–	77	15	–	92	–	–	69	–	–	0	–
Yu N 2020.3.24 [22]	2020.1.1–2020.2.8	7	32	0	86	14	71	100	29	0	100	–	0	14	–
Zhu H 2020.2 [23]	2020.1.20–2020.2.5	9	30	–	89	44	–	100	0	33	78	16	11^(c)^	0	–

^(a)^ The patients are from American hospitals in the two literatures, and others are from Chinese hospitals

^(b)^ 1 case was stillbirth

^(c)^ 1 case was neonatal death

### Assessment of quality

We evaluated the quality of the fourteen included documents according to the IHE case series methodological quality evaluation tool. Thirteen articles had quality values ranging from 45 to 65%, all of which were of low quality because the values were lower than 70%. Only one article had a quality value that reached 70% and was considered to be of acceptable quality. These articles were all retrospective studies with few cases and without control groups, interventions or blind methods, so they were rated as low quality. However, these are the only published documents at present, which necessitated their inclusion. The literature quality assessment is shown in Table 2.

**Table 2:** Literature quality assessment with IHE case series methodological quality evaluation tool

	Research purpose	Research population	Intervention and joint intervention	Outcome measures	Statistical analysis	Results and conclusions	Conflict of interest and funding sources	New entry	Total	Percentage (%)
Breslin N 2020.4.9 [10]	1	5	0	2	1	4	1	0	14	70
Chen H 2020.2.12 [11]	1	4	0	2	1	3	1	0	12	60
Chen R 2020.3.16 [12]	1	4	1	3	0	4	0	0	13	65
Chen S 2020.3.28 [13]	1	4	0	2	0	3	1	0	11	55
Khan S 2020.4.8 [14]	1	4	0	2	0	3	1	0	11	55
Li N 2020.3.30 [15]	1	4	0	2	1	4	1	0	13	65
Liu D 2020.3.7 [16]	1	4	0	2	1	4	0	0	12	60
Liu H 2020.3.11 [17]	1	4	0	2	1	4	1	0	13	65
Liu Y 2020.2.27 [18]	1	5	0	2	0	3	0	0	11	55
Sutton D 2020.4.13 [19]	1	3	0	2	0	3	0	0	9	45
Wu X 2020.4.8 [20]	1	4	0	2	1	4	1	0	13	65
Yang H 2020.4.12 [21]	1	4	0	2	1	4	1	0	13	65
Yu N 2020.3.24 [22]	1	4	0	2	1	4	1	0	13	65
Zhu H 2020.2 [23]	1	5	0	2	0	3	1	0	12	60

### Quantitative data synthesis

Because of the high heterogeneity of this study, we used a random effects model. The meta-analysis showed the following results: the incidence of severe case or death was 12, 95% CI: 0.03–0.20, *I*^*2*^ = 0%, *P* = 0.006; the incidence of fever was 51, 95% CI: 0.35–0.67, *I*^*2*^ = 89%, *P* < 0.00001; the incidence of cough was 31, 95% CI: 0.23–0.39, *I*^*2*^ = 38%, *P* < 0.00001; the incidence of lymphopenia was 49, 95% CI: 0.29–0.70, *I*^*2*^ = 83%, *P* < 0.00001; the incidence of positive CT findings was 71, 95% CI: 0.49–0.93, *I*^*2*^ = 90%, *P* < 0.00001; the incidence of coexisting disorders was 33, 95% CI: 0.21–0.44, *I*^*2*^ = 70%, *P* < 0.00001; the incidence of preterm labor was 23, 95% CI: 0.14–0.32, *I*^*2*^ = 21%, *P* < 0.00001; the incidence of caesarean section was 65, 95% CI: 0.42–0.87, *I*^*2*^ = 90%, *P* < 0.00001; the incidence of fetal distress was 29, 95% CI: 0.08–0.49, *I*^*2*^ = 68%, *P* = 0.007; the incidence of neonatal asphyxia or neonatal death or stillbirth was 9, 95% CI: − 0.03-0.21, *I*^*2*^ = 0%, *P* = 0.14; the incidence of neonatal infection was 12, 95% CI: − 0.01-0.26, *I*^*2*^ = 0%, *P* = 0.06; and SARS-CoV-2 testing of breast milk was only mentioned in the study by Chen H (2020.2.12), and the incidence was 0, which cannot be calculated by meta-analysis.

In summary, the *P* values of neonatal asphyxia or neonatal death or stillbirth and neonatal infection were both greater than 0.05, which were not statistically significant. We also could not calculate the incidence of a positive SARS-CoV-2 testing in breast milk. Otherwise, the *P* values in the remaining indicators were all less than 0.05 and were statistically significant. The most common clinical features were positive CT findings (71%), caesarean section (65%), and fever (51%), followed by lymphopenia (49%), cough (31%) and severe case or death (12%). Adverse pregnancy outcomes included coexisting disorders (33%), fetal distress (29%) and preterm labor (23%), in descending order. Among these indicators, the *I*^*2*^ value of severe cases or deaths was 0%, which indicated low heterogeneity. Although the indicators mentioned above refer to 10 studies, the incidences in eight documents were all 0, and there were only two non-zero indicator data points. The *I*^*2*^ value of preterm labor was 21%, which indicated low heterogeneity. The *I*^*2*^ value of cough was 38%, which indicated medium heterogeneity, and the remaining *I*^*2*^ values of indicators ranged from 68 to 90%, which indicated high heterogeneity.

**Fig. 2:**
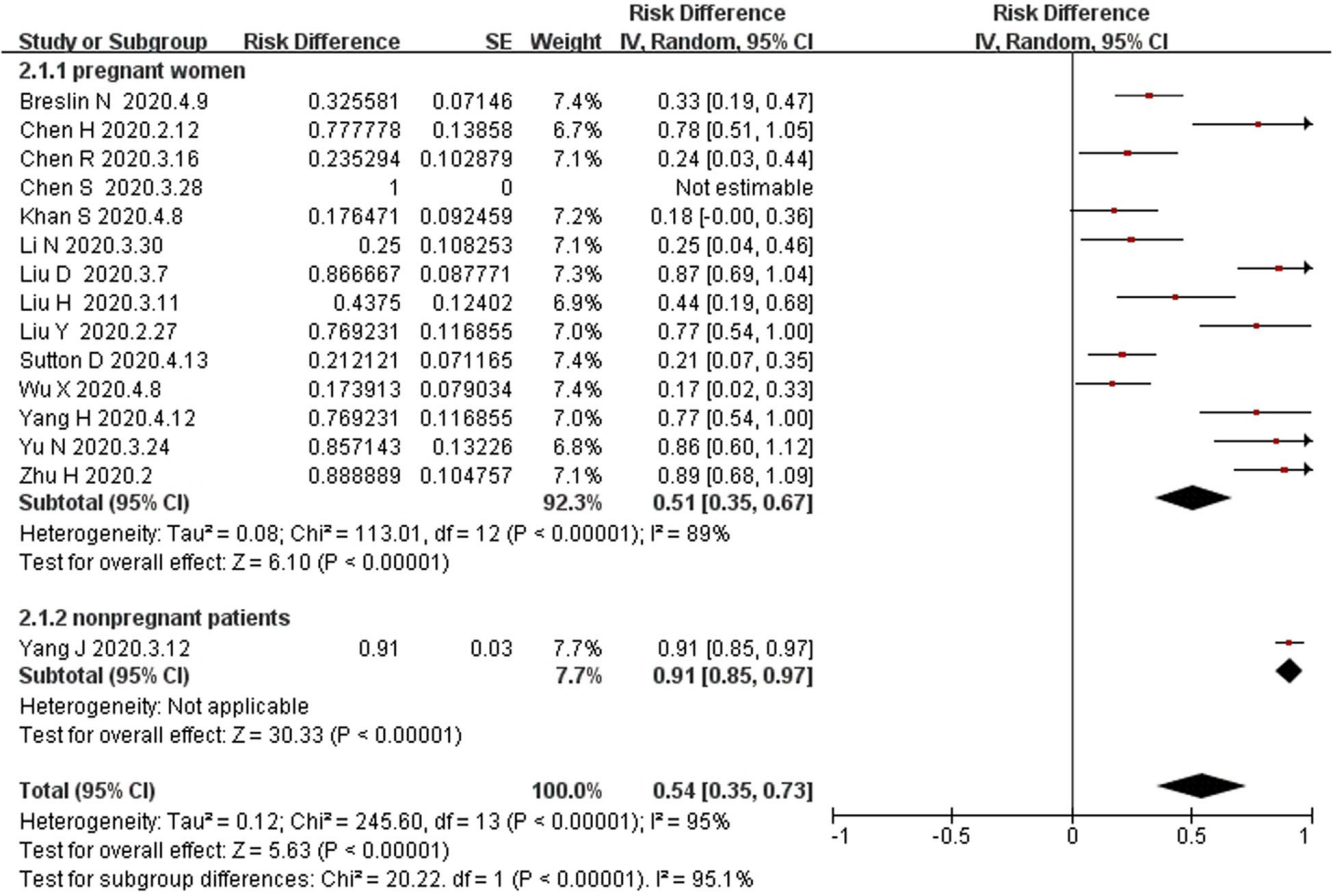
The forest plot of subgroup analysis of fever

**Fig. 3:**
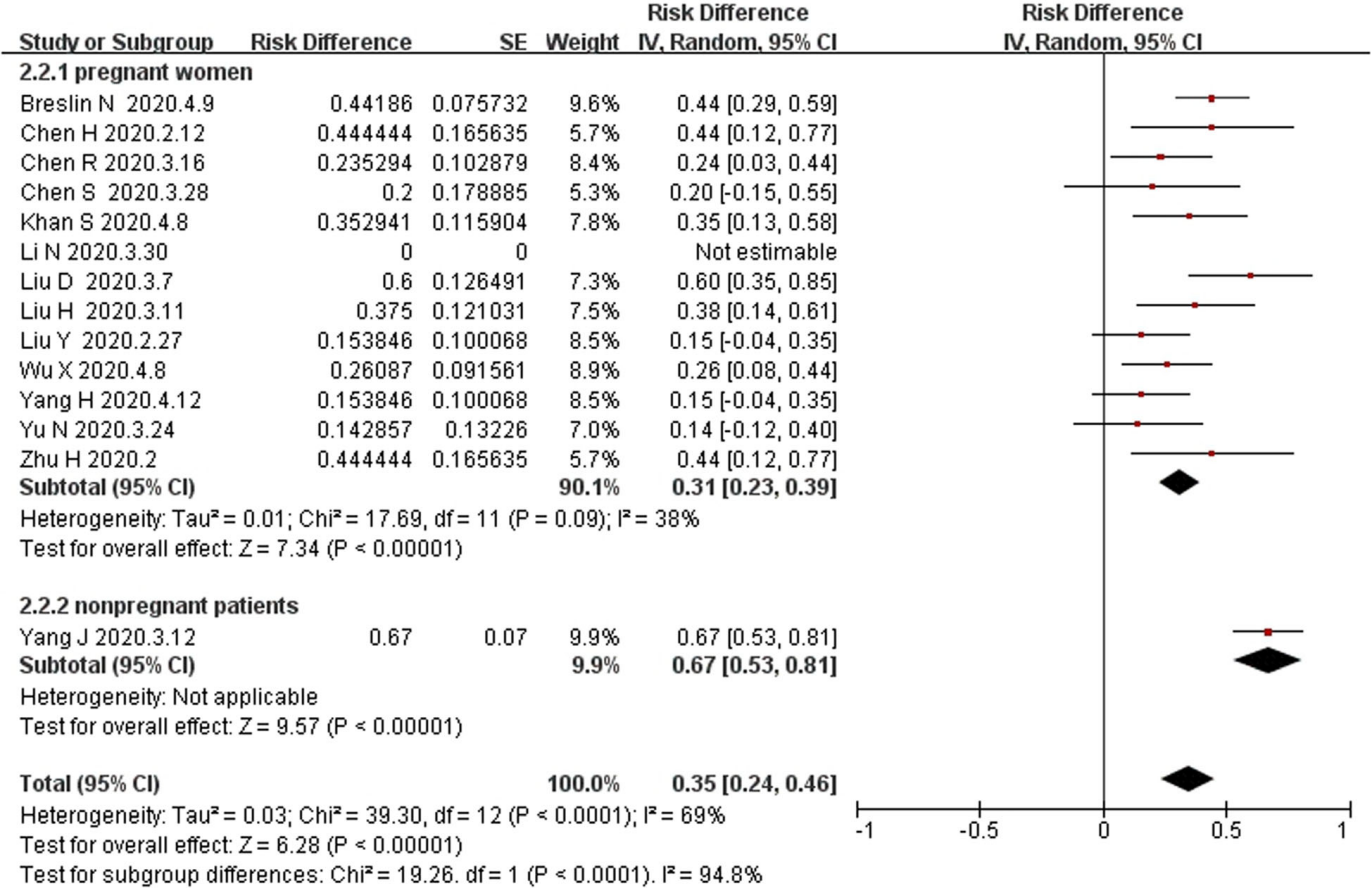
The forest plot of subgroup analysis of cough

Furthermore, we carried out a subgroup analysis based on the data from the fourteen retrospective analyses of COVID-19 infection in the pregnant women above and one meta-analysis of the epidemiology of all the patients COVID-19 [24]. All the patients were divided into two subgroups, namely, pregnant women and non-pregnant patients. In the fifteen articles, only two indices, i.e., fever and cough, were coincident, and were analyzed in subgroups. The results were as follows. The incidence of fever in the pregnant women was 51%, which was significantly lower than the 91% fever incidence in the non-pregnant patients (*P* < 0.00001). The incidence of cough in the pregnant women was also significantly lower than that in the non-pregnant patients (31% vs 67%, *P* < 0.0001). The forest plot of the subgroup analysis is illustrated in Figs. 2 and 3.

### Risk of publication bias

**Fig. 4:**
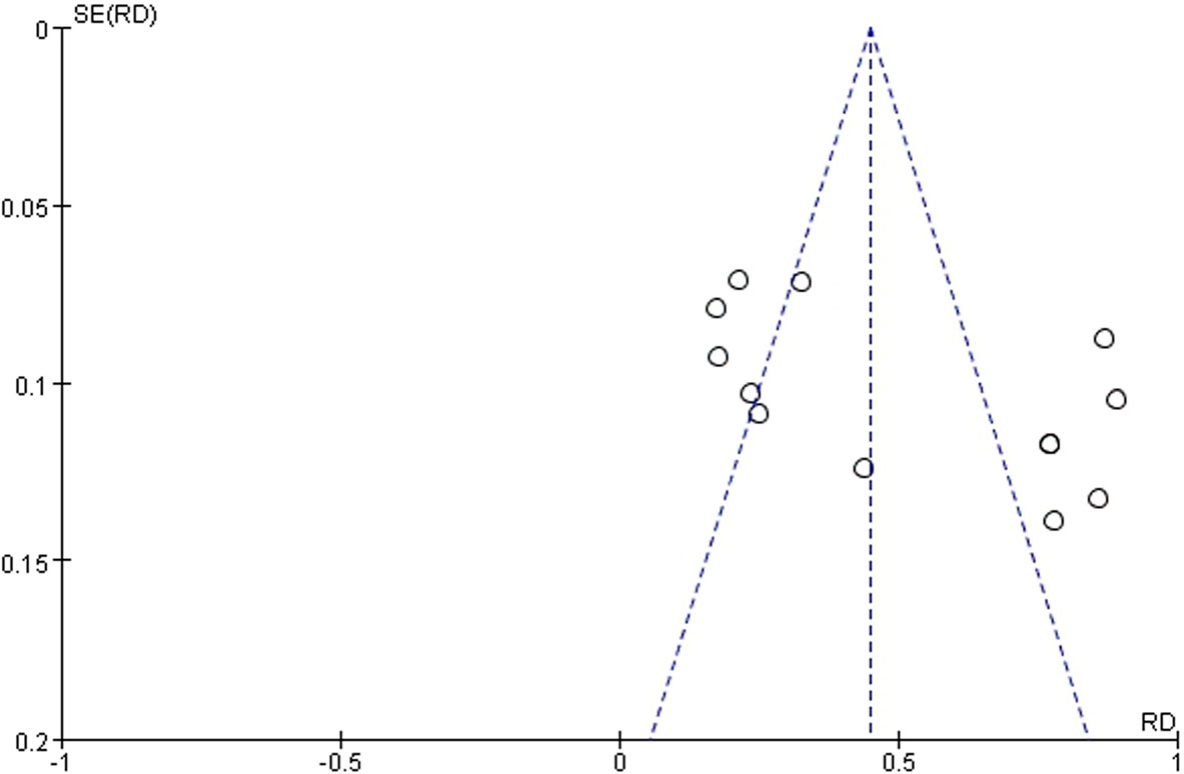
Funnel plot of fever

**Fig. 5:**
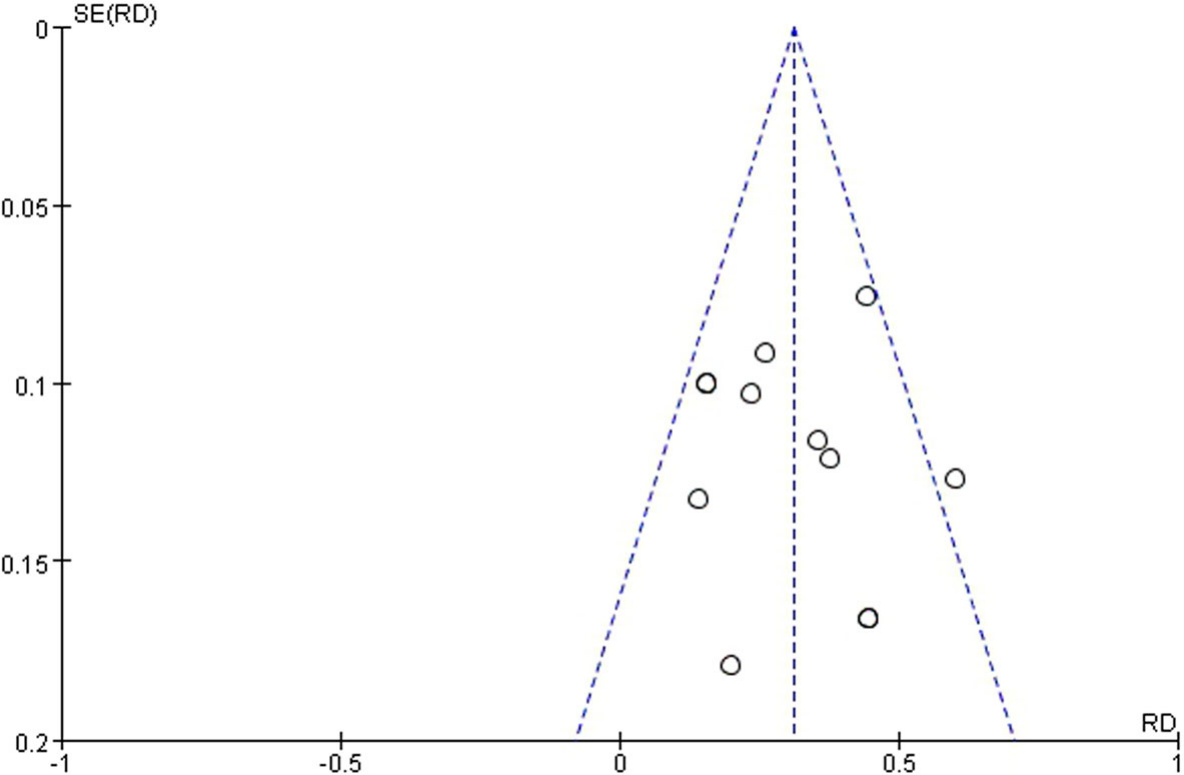
Funnel plot of cough

The funnel plots of fever, cough, positive CT findings and coexisting disorders were symmetric, which meant that these indicators may not have publication bias. In contrast, the funnel plots of severe case or death, lymphopenia, preterm labor, caesarean section, fetal distress, neonatal asphyxia or neonatal death or stillbirth and neonatal infection were asymmetric, which meant that the indicators above may have publication bias. Since there was only 1 article about SARS-CoV-2 testing of breast milk, it was meaningless to draw a funnel plot; therefore, publication bias was not evaluated. The funnel plots of fever and cough are shown in Figs. 4 and 5.

## Discussion

### Main findings

The cases discussed in this article involved 160 pregnant women with COVID-19 in China and 76 pregnant women with COVID-19 in America. The pooled results of this meta-analysis showed that among the pregnant women with COVID-19, 71% had positive CT findings, 65% had a caesarean section, 51% had fever, 49% had lymphopenia in laboratory examination, 33% had coexisting disorders, 31% had cough, 29% had fetal distress, 23% had preterm labor and 12% had severe cases or died. Pregnant women with COVID-19 had significantly lower rates of fever and cough than non-pregnant patients with COVID-19.

### Strengths and limitations

Currently, there are only meta-analyses of the epidemiology of typical patients infected with COVID-19, and there are few meta-analyses to explore the clinical features and outcomes of pregnant women with COVID-19. This study is helpful to formulate clinical treatment strategies for pregnant women with COVID-19.

The disadvantage of this study was the small sample size and the general quality of the included documents, which lowered the credibility of the meta-analysis results. In addition, all the included articles were retrospective case analyses without control groups, which also biased the results. Furthermore, the funnel plots showed that most indicators may have publication bias. Most of the included patients were Chinese, and the others were American. There are few reports describing the cases outside the two regions. Further research on pregnant women with COVID-19 worldwide is needed.

### Interpretation

We referred to the normal population the non-pregnant group to distinguish it from the pregnant group in the subgroup analysis. The subgroup analysis between pregnant women with COVID-19 and non-pregnant patients with COVID-19 showed that the incidences of fever and cough in pregnant women with COVID-19 (51, 31%) were lower than those in normal people (91, 67%), which may be due to the changes in the immune system of pregnant women, and further research is needed. The available data do show no increased or even lower maternal mortality rate after infection [25], but it does not mean that pregnancy is a protective factor for severe infection. It was reported that severe infection mostly occurred in the elderly (> 60 years old), patients with basic diseases, such as diabetes, obesity, hypertension, coronary heart disease, cerebrovascular diseases, and other chronic diseases [26–30], as well as those who did not receive timely treatment or delay treatment [31]. Pregnant women are usually younger without primary diseases. Besides, pregnant women are usually more likely to receive attention after the onset of the disease (pregnant women themselves, family members, and medical staff), with fewer delays in treatment. All these can explain the low fatality rate of infected pregnant women. There is no evidence that pregnancy can alleviate the disease yet, which needs further study. Aya Mohr-Sasson et al. compared clinical characteristics between pregnant women and non-pregnant women. The study showed that there were no statistical differences in clinical features such as respiratory symptoms and fever between the two groups [32]. It was reported that male patients were likely to develop more severe symptoms and have higher prevalence rates and mortality rates than female patients [33–35]. Zeng F et al. showed that compared with male patients, more female patients were generating a relatively high level of SARS-CoV-2 IgG antibody in severe cases, and the IgG antibody presented a stronger production in female patients in disease early phase [36]. It may be the reason why the clinical characteristics of the pregnant group in this study are better than those of the non-pregnant group. A study from the China CDC showed that 80.9% of Chinese patients were considered to be asymptomatic or to have mild pneumonia [37]. Desmond Sutton et al. showed that of the 215 pregnant women who gave birth at the New York-Presbyterian Allen Hospital and Columbia University Irving Medical Center, 29 (87.9%) of the 33 patients who were positive for SARS-CoV-2 testing had no symptoms of COVID-19 at the time of admission [19]. The fact that the asymptomatic rate in the infected general population in China is lower than the rate in infected pregnant women in the New York Medical Center seems to support this conclusion in this study. These findings suggest that the SARS-CoV-2 testing should be universally administered in high-risk areas to improve the isolation of asymptomatic infected individuals. This result is different from the finding that pregnant women infected with SARS-CoV have a worse prognosis than ordinary people infected with SARS-CoV [38]. It is possible that in pregnant women, the clinical outcome of COVID-19 infection is better than that of SARS-CoV. Yan et al. confirmed that the current mortality rate of COVID-19(2%) is significantly lower than that of SARS (9.6%), which may indicate that SARS is more pathogenic and lethal than COVID-19; thus, pregnant women with COVID-19 infection had better outcomes than those with SARS-CoV [39]. However, our finding that pregnant women with COVID-19 had better clinical features might be biased owing to the relatively small sample included in this meta-analysis.

A meta-analysis showed that the CT positive rate of COVID-19 in the normal population was 89.76% [40], which was more than the 71% positive rate in this paper. This finding also corresponded to the conclusion above that the clinical features of pregnant women with COVID-19 were superior to those of the general population. The incidence of positive CT findings was the highest among the selected indicators. Shital J. Patel et al. confirmed that chest CT was considered a low-dose examination, provided the foetus was excluded from the primary beam, and the estimated radiation doses were too low to induce foetus neurologic deficits during any trimester of pregnancy [41]. It seemed that chest CT was suitable for routine screening of patients. However, there was a large percentage of pregnant women with asymptomatic infections (87.9%) [19]. If chest CT is used for routine screening, it means that almost all pregnant women need to undergo chest CT. In addition, the WHO defines screening as the presumptive identification of unrecognized disease in an apparently healthy, asymptomatic population by means of tests, examinations or other procedures that can be applied rapidly and easily to the target population [42]. Consequently, it is not appropriate to perform chest CT as a screening tool for pregnant women with COVID-19. We recommend using chest CT as the routine examination for suspected cases.

The rate of preterm labor in normal pregnant women who are healthy and not infected with any virus worldwide is approximately 11% [43], which is lower than the result in this article (23%). The possible reason for the higher rate is that women in the third trimester of pregnancy induce delivery early after becoming ill with COVID-19 to proceed with further treatment. Most of these women choose early delivery by caesarean section to avoid a prolonged labor, which may worsen COVID-19 for pregnant women [44] and increase the risk of infection for the medical staff [45]. Chen R et al. confirmed that both epidural anesthesia and general anesthesia were safe and effective for women with COVID-19 during caesarean section [12].

Because the *P* value was greater than 0.05, the rate of neonatal COVID-19 infection should not be considered. Wang S et al. reported the first case in China in which a mother with COVID-19 gave birth to an infected baby on February 2, 2020 [46], and the instant SARS-CoV-2 nucleic acid tests of the umbilical cord blood and placenta were both negative. There were 3 infected neonates in the included literature. Khan S. et al. reported that the swab samples tested within 24 h after delivery were positive in two neonates, and intrauterine tissue samples such as placenta, cord blood or amniotic fluid were not tested [14]. Yu N et al. reported that the nucleic acid test for the throat swab of one neonate was positive at 36 h after birth [22]. Without testing the intrauterine tissue samples, we could not confirm whether the SARS-COV-2 infection in the neonate was the result of intrauterine transmission. Two studies also showed that the test for SARS-CoV-2-specific antibodies (IgG and IgM) in neonatal serum samples could be evidence of vertical transmission [47, 48]. Other literature revealed that almost all the other new-borns from infected women tested negative for SARS-CoV-2 [10–13, 15–21, 23, 49–52]. Wang C et al. summarized that there was currently no evidence for intrauterine infection caused by vertical transmission in women with COVID-19 during the third trimester of pregnancy, but it was uncertain whether there could be a risk of vertical transmission when the COVID-19 infection occurs in the first or second trimester or when there was a long clinical manifestation-to-delivery interval [53]. Therefore, we must remain alert to the possibility of vertical transmission.

## Conclusion

The incidence of fever, cough and positive CT findings in pregnant women with COVID-19 is less than that in the normal population with COVID-19. The rate of preterm labor in normal pregnant women worldwide who are healthy and not infected with any virus is lower than that in pregnant women with COVID-19. There is currently no evidence that COVID-19 can spread through vertical transmission. The conclusions above are possibly helpful to formulate clinical treatment strategies for pregnant women with COVID-19.

## Data Availability

The datasets used and/or analyzed during the current study are available from the corresponding author on reasonable request.

## Abbreviations

SARS-CoV: .
WHO: .
2019-nCoV: .
SARS-CoV-2: .
COVID-19: .
the MOOSE checklist: .
EQUATOR Reporting Guidelines: .
IHE case series methodological quality evaluation tool: .
95% CI: .
